# Shifts in body mass index category with tirzepatide and associated changes in cardiometabolic risk factors in people with obesity: a post hoc analysis from the SURMOUNT-1 and SURMOUNT-4 trials

**DOI:** 10.1016/j.ajpc.2026.101454

**Published:** 2026-02-25

**Authors:** Naveed Sattar, Clare J. Lee, Reshmi Srinath, Philip R. Schauer, Hui Wang, Beverly Falcon, Xuanyao He, Adam Stefanski, Arian Plat

**Affiliations:** a School of Cardiovascular and Metabolic Health, University of Glasgow, 126 University Place, Glasgow, G12 8TA, United Kingdom; b Eli Lilly and Company, Lilly Corporate Center, Indianapolis, IN 46285, USA; c Mount Sinai Diabetes Center, Icahn School of Medicine at Mount Sinai, 5 East 98th Street, 3rd Floor, New York, NY, 10029, USA; d Pennington Biomedical Research Center, Louisiana State University, 6400 Perkins Rd, Baton Rouge, LA, 70808, USA; e Techdata Service Company, 700 American Ave Ste 102, King of Prussia, PA, 19406, USA

**Keywords:** Tirzepatide, Obesity, Overweight, Cardiovascular risk, Body mass index

## Abstract

**Objective:**

Obesity is a chronic disease that results in increased morbidity and mortality if left untreated. Tirzepatide is a glucose-dependent insulinotropic polypeptide/glucagon-like peptide-1 receptor agonist approved in the United States for the treatment of type 2 diabetes, obesity, and obstructive sleep apnea. These post hoc analyses assessed the cardiometabolic risk factors of participants with obesity or overweight treated with tirzepatide who shifted to a lower body mass index (BMI) category.

**Methods:**

Shifts in BMI categories (<25, 25 to <30, 30 to <35, 35 to <40, and ≥40 kg/m^2^) from baseline to Week 72 (SURMOUNT-1) and Week 88 (SURMOUNT-4) were assessed. BMI shifts were classified as improved (shift to ≥1 lower category) or not improved (no change/shift to a higher category). Changes from baseline in weight, waist circumference, fasting insulin, fasting glucose, glycated hemoglobin, vitals, blood pressure, and lipid profile were measured.

**Results:**

Improvement in BMI category from baseline occurred in 81.8 % of participants treated with tirzepatide in SURMOUNT-1 and 91.6 % in SURMOUNT-4. Among these participants, mean BMI reductions were greater than in those without improvement (SURMOUNT-1: -8.90 vs -3.65 kg/m^2^; SURMOUNT-4: -10.47 vs -5.20 kg/m^2^). In the improved BMI category, tirzepatide treatment showed significant improvement in cardiometabolic parameters in both studies.

**Conclusion:**

In these post hoc analyses, the majority of tirzepatide-treated participants with obesity or overweight shifted to lower BMI categories. Tirzepatide treatment was associated with significantly improved cardiometabolic risk factors in participants who shifted to a lower BMI category, which may positively impact long-term cardiovascular outcomes.

**Figure:**
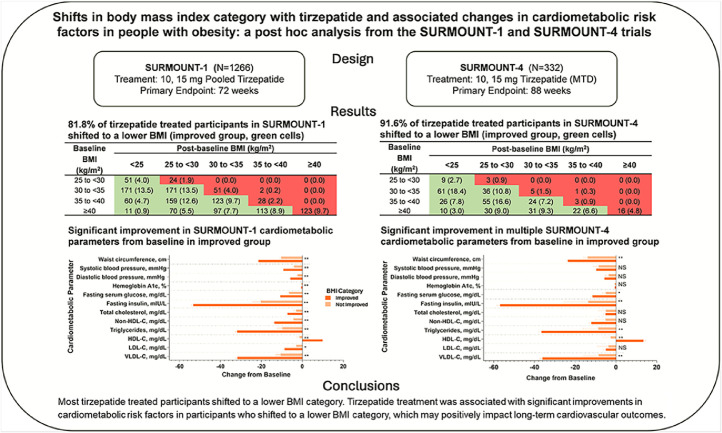
Central Illustration. This post hoc analysis of two clinical trials (SURMOUNT-1 and SURMOUNT-4) assessed cardiometabolic risk factors in participants with obesity or overweight treated with tirzepatide, who shifted to a lower BMI category. Percent change from baseline in fasting insulin and lipids is calculated using log transformation and presented as estimate (SE). Changes from baseline are reported for all other parameters. *p<0.01, **p<0.001 improved vs not improved. BMI = body mass index; HDL-C = high-density lipoprotein cholesterol; LDL-C = low-density lipoprotein cholesterol; MTD = maximum tolerated dose; N = total number of participants; NS = not significant; SE = standard error; VLDL-C = very-low-density lipoprotein cholesterol.

## 1 Introduction

Obesity is characterized by excess adiposity, and individuals may or may not exhibit an abnormal distribution or function of adipose tissue [1]. The causes of obesity are multifactorial and not yet fully understood [2]. The worldwide prevalence of obesity—defined as having a body mass index (BMI) of 30 kg/m^2^ or higher—has doubled between 1990 and 2022 [3]. In 2022, 43 % of adults worldwide were overweight (BMI ≥25 kg/m^2^), and 16 % had obesity [3]. Having overweight and obesity increases the risk of all-cause mortality and other health problems, including hypertension, dyslipidemia, obstructive sleep apnea, and cardiovascular diseases such as heart failure with preserved ejection fraction [4,5].

Current guidelines recommend a weight loss of at least 5 % in people living with obesity and recognize that larger, sustained weight losses (≥10 %) likely confer greater benefits, including prevention of type 2 diabetes (T2D), potential improvement in long-term cardiovascular outcomes, and reduced mortality [6,7].

The relationship between obesity and cardiovascular disease is only partially understood due to multiple overlapping risk factors [2]. However, compelling data document an association between increased BMI and worsening cardiometabolic risk factors [8,9]. Additionally, there is evidence that an approved treatment for obesity reduces the risk of atherosclerotic cardiovascular disease [10]. Yet, there are limited data examining patterns of change in BMI categories and their impact on cardiometabolic risk factors in adults with overweight or obesity.

In the SURMOUNT-1 and SURMOUNT-4 trials, treatment with once-weekly glucose-dependent insulinotropic polypeptide and glucagon-like peptide-1 receptor agonist tirzepatide resulted in superior body weight reductions of 20.9 % (SURMOUNT-1) to 25.3 % (SURMOUNT-4) in people living with obesity [8,11]. The present post hoc analyses assessed shifts in BMI categories following tirzepatide treatment and their association to cardiometabolic risk factors in adults with overweight or obesity from the SURMOUNT-1 and SURMOUNT-4 trials.

## 2 Materials and methods

### 2.1 Participants and study design

The study designs, full inclusion and exclusion criteria, and primary results of the SURMOUNT-1 and SURMOUNT-4 trials have been previously reported [8,11]. Briefly, the phase 3 SURMOUNT clinical program for weight management consisted of randomized controlled clinical studies lasting 72 to 88 weeks. These studies assessed tirzepatide doses of 5 mg, 10 mg, and 15 mg, or maximum tolerated dose (MTD) of 10 mg and 15 mg, in adults with obesity (BMI ≥30 kg/m^2^) or overweight (BMI ≥27–30 kg/m^2^) with at least one weight-related comorbid condition (hypertension, dyslipidemia, obstructive sleep apnea, or cardiovascular disease) without T2D. Only data from tirzepatide-treated participants were included in the analysis.

**Fig. 1:**
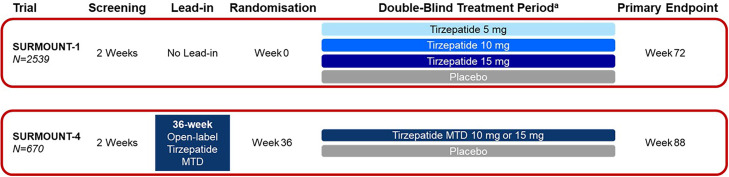
Study designs for SURMOUNT-1 and SURMOUNT-4. ^a^Adjunct to a reduced calorie diet and increased physical activity. Notes: SURMOUNT-1, and -4 included participants with obesity (BMI ≥30 kg/m^2^) or overweight (BMI ≥27 kg/m^2^) with ≥1 weight-related condition, excluding diabetes. MTD = maximum tolerated dose; N = number of participants in the population.

All clinical trials were conducted in accordance with the International Council for Harmonisation of Technical Requirements for Pharmaceuticals for Human Use and the Declaration of Helsinki. All participants provided signed informed consent, and protocols were approved by local ethical review boards. The program was sponsored by Eli Lilly and Company (Indianapolis, IN, USA). The SURMOUNT clinical trials assessed in these analyses were SURMOUNT-1 (NCT04184622) and SURMOUNT-4 (NCT04660643) (Fig. 1).

### 2.2 Endpoints

In these post hoc analyses of SURMOUNT-1 and SURMOUNT-4 participants treated with tirzepatide, BMI shifts were classified as improved (shift to ≥1 lower category) or not improved (no change in category or shift to ≥1 higher category). Shifts between these BMI categories (<25 kg/m^2^, 25 to <30 kg/m^2^, 30 to <35 kg/m^2^, 35 to <40 kg/m^2^, and ≥40 kg/m^2^) from baseline (Week 0) to Week 72 in SURMOUNT-1 were assessed. For comparability between the two trials, the 10 mg and 15 mg arms of SURMOUNT-1 were pooled for this analysis, and the 5 mg arm of SURMOUNT-1 was excluded. For SURMOUNT-4, BMI shifts from lead-in baseline to the primary endpoint (Week 88) in participants treated with tirzepatide MTD (10 mg or 15 mg) were determined. Changes from baseline in weight, waist circumference, fasting insulin, fasting glucose, glycated hemoglobin (HbA1c), vitals, blood pressure, and lipid profile were assessed for both trials [8,11].

### 2.3 Statistical analysis

#### Post hoc analysis from SURMOUNT-1

Shift in BMI from baseline to Week 72 was assessed using the efficacy analysis set, which includes data obtained during the treatment period from the modified intent-to-treat population, excluding data after discontinuation of the study drug (last dose date + 7 days) (*N* =1254). The modified intent-to-treat population is defined as all randomly assigned participants who received at least one dose of study drug. Missing BMI values at Week 72 were imputed using the last observation carried forward. Cardiometabolic markers were analyzed for (pooled) 10 mg and 15 mg tirzepatide treated participants using a mixed model for repeated measures (MMRM). The MMRM included BMI improved and not improved group, visit, BMI-group-by-visit-interaction, prediabetes status at randomization, pooled country, and sex as fixed effects, and baseline body weight as a covariate. An unstructured covariance structure modeled relationship of within-patient errors. The association of baseline parameters with BMI shifts was assessed with an initial univariate analysis, followed by stepwise multivariate logistic regression with entry into the model set at p<0.05, based on the p-value from the univariate analysis.

#### Post hoc analysis from SURMOUNT-4

Shift in BMI category from Week 0 to Week 88 was assessed in participants randomized to tirzepatide MTD (N=332) using the same method as described for SURMOUNT-1. The MMRM model was adjusted for pooled country, sex, MTD dose at randomization, weight loss at randomization (<10 %, ≥10 %), and baseline values at lead-in. The association of BMI shifts with baseline parameters were assessed using the same method described for SURMOUNT-1.

## 3 Results

### 3.1 Participants

The baseline demographics and clinical characteristics of participants in the SURMOUNT-1 and SURMOUNT-4 trials have been reported previously [8,11]. Select baseline demographics based on end-of-study BMI category shifts for SURMOUNT-1 and SURMOUNT-4 are presented in Table 1.

**Table 1:** Demographic and clinical characteristics of SURMOUNT-1 and SURMOUNT-4 participants based on end-of-study BMI shift categories.

	SURMOUNT-1 BMI Shift Category at Week 72, Pooled Tirzepatide (10 mg/15 mg)^a^ Total N=1254			SURMOUNT-4 BMI Shift Category at Week 88, Tirzepatide MTD (10 mg or 15 mg) Total N=332		
Baseline Parameter	Improved (N=1026)	Not Improved (N=228)	p-value	Improved (N=304)	Not Improved (N=28)	p-value
Age, years	44.9 (12.2)	44.0 (12.8)	0.322	48.5 (12.6)	45.6 (14.0)	0.246
Female, n ( %)	724 (70.6)	123 (53.9)	<0.001	220 (72.4)	14 (50.0)	0.013
Race, n ( %)						
Asian	114 (11.1)	23 (10.1)	0.660	23 (7.6)	3 (10.7)	0.831
Black/African American	73 (7.1)	22 (9.6)		37 (12.2)	2 (7.1)	
Native Hawaiian/Pacific Islander	5 (0.5)	0		1 (0.3)	0	
White	728 (71.0)	158 (69.3)		238 (78.3)	23 (82.1)	
Multiple	12 (1.2)	2 (0.9)		5 (1.6)	0	
Ethnic group						
Hispanic/Latino	482 (47.0)	108 (47.4)	0.879	132 (43.4)	8 (28.6)	0.292
Education, years	14.0 (3.8)	13.9 (4.3)	0.597	14.6 (4.5)	14.1 (4.8)	0.648
Duration of obesity, years	14.6 (10.9)	15.7 (11.2)	0.165	15.7 (12.0)	17.3 (14.4)	0.500
Weight, kg	102.1 (19.3)	121.8 (31.0)	<0.001	105.1 (19.2)	125.1 (28.8)	<0.001
BMI, kg/m^2^	37.1 (5.4)	43.0 (10.0)	<0.001	37.7 (5.5)	44.1 (9.7)	<0.001
Waist circumference, cm	112.4 (13.4)	124.7 (20.5)	<0.001	114.0 (12.8)	124.2 (20.0)	<0.001
HbA1c, %	5.5 (0.4)	5.6 (0.4)	0.052	5.6 (0.4)	5.6 (0.3)	0.513
Systolic blood pressure, mmHg	123.0 (12.9)	124.6 (12.8)	0.097	126.6 (13.7)	129.8 (9.8)	0.234
Diastolic blood pressure, mmHg	79.6 (8.3)	79.5 (7.9)	0.860	80.8 (8.2)	80.9 (8.4)	0.957
Pulse rate, bpm	71.8 (9.5)	73.5 (10.7)	0.015	72.2 (9.7)	69.5 (9.0)	0.159
eGFR, mL/min/1.73 m^2^	97.9 (17.6)	100.4 (19.5)	0.060	97.5 (17.0)	99.3 (20.3)	0.603
Hypertension, n ( %)^b^	321 (31.3)	89 (39.0)	0.024	105 (34.5)	13 (46.4)	0.208
Dyslipidemia, n ( %)^b^	301 (29.3)	64 (28.1)	0.703	104 (34.2)	9 (32.1)	0.825
Obstructive sleep apnea, n ( %)^b^	70 (6.8)	24 (10.5)	0.055	36 (11.8)	3 (10.7)	0.859
Atherosclerotic CVD, n ( %)^b^	32 (3.1)	9 (3.9)	0.525	15 (4.9)	3 (10.7)	0.196

Data are presented as mean (SD), unless otherwise indicated, based on the randomized population. Baseline in SURMOUNT-1 refers to the baseline at Week 0 randomization; baseline in SURMOUNT-4 refers to the lead-in period Week 0 baseline. The p-value for overall treatment effect was computed using the χ^2^ test for categorical variables and using ANOVA for continuous variables.

ANOVA = analysis of variance; BMI = body mass index; bpm = beats per minute; CVD = cardiovascular disease; eGFR = estimated glomerular filtration rate; HbA1c = glycated hemoglobin; MTD = maximum tolerated dose; N = number of participants in the given population; n = number of participants meeting the criteria of the parameter; SD = standard deviation

a Data from the 10 mg and 15 mg dose groups in SURMOUNT-1 were pooled for this analysis.

b The majority of cases were “not reported”.

### 3.2 Shifts in BMI category among participants treated with tirzepatide

**Fig. 2:**
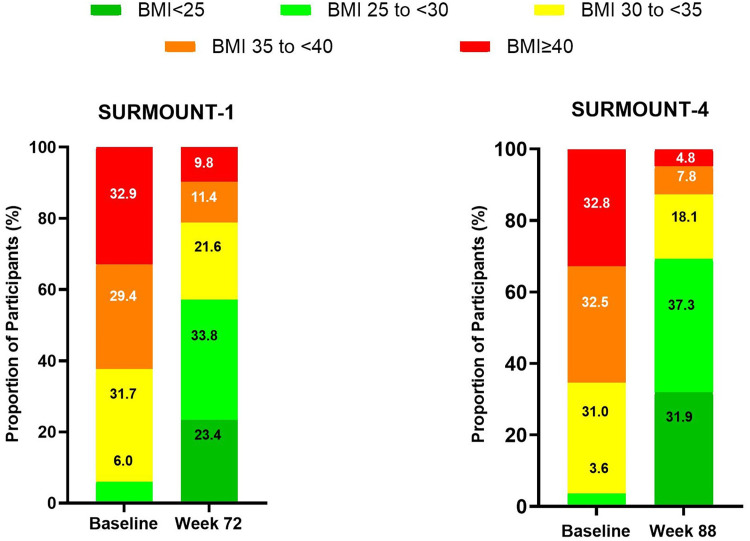
Proportion of participants treated with tirzepatide in each BMI category at baseline and Week 72 (SURMOUNT-1) and baseline and Week 88 (SURMOUNT-4). Numbers within each column boxes are the percentage of participants treated with tirzepatide in that BMI category. SURMOUNT-1 = pooled 10 mg/15 mg; SURMOUNT-4 = maximum tolerated dose. Baseline in SURMOUNT-4 is the lead-in baseline at Week 0. BMI = body mass index.

**Fig. 3:**
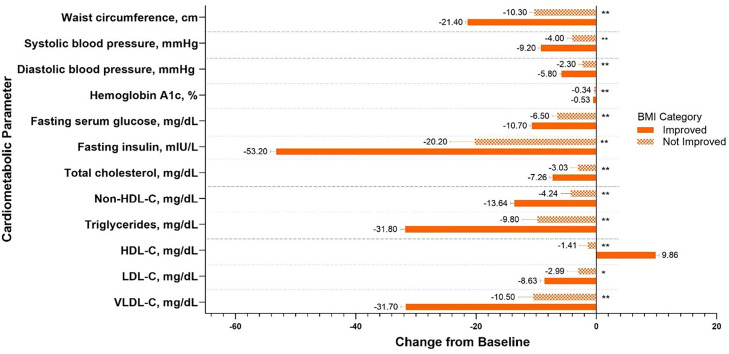
Change from baseline to Week 72 in cardiometabolic parameters in the improved and not improved BMI categories for participants treated with tirzepatide 10 mg and 15 mg in SURMOUNT-1. Data are presented as LSM (SE) change from baseline at Week 72 from the randomized population. *p<0.01, **p<0.001 improved vs not improved. Percent change from baseline in fasting insulin and lipids is reported and calculated using log transformation and presented as estimate (SE). Change from baseline is reported for other parameters. Data from the 10 mg and 15 mg tirzepatide dose groups were combined. BMI = body mass index; HDL-C = high-density lipoprotein cholesterol; LDL-C = low-density lipoprotein cholesterol; LSM = least squares mean; SE = standard error; VLDL-C = very-low-density lipoprotein cholesterol.

**Fig. 4:**
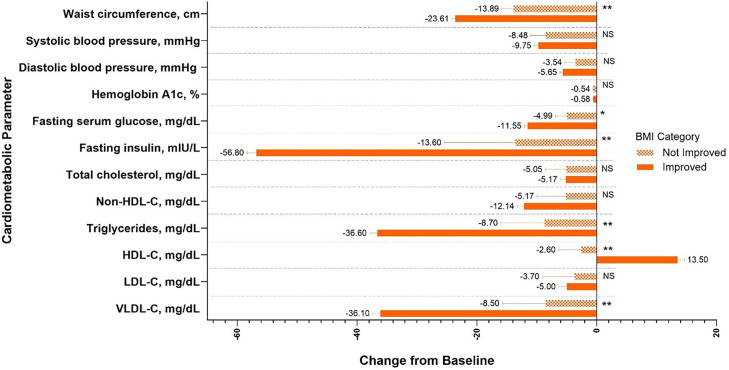
Change from baseline to Week 88 in cardiometabolic parameters in the improved and not improved BMI categories in participants treated with the MTD of tirzepatide in SURMOUNT-4. Data are presented as LSM (SE) change from baseline at Week 72 from the randomized population. *p < 0.01, **p < 0.001 improved vs not improved. Percent change from baseline in fasting insulin and lipids is reported and calculated using log transformation and presented as estimate (SE). Change from baseline is reported for other parameters. BMI = body mass index; HDL-C = high-density lipoprotein cholesterol; LDL-C = low-density lipoprotein cholesterol; LSM = least squares mean; MTD = maximum tolerated dose; NS = not significant; SE = standard error; VLDL-C = very-low-density lipoprotein cholesterol.

In both SURMOUNT-1 and SURMOUNT-4, tirzepatide treatment resulted in shifts to a lower BMI category compared to baseline (Figs. 2, 3, and 4). In SURMOUNT-1, 81.8 % of participants treated with tirzepatide (pooled 10 mg/15 mg) shifted at least one BMI category lower (Table 1, Table 2), and the mean BMI change from baseline was -8.90 kg/m^2^ in the improved group versus -3.65 kg/m^2^ in the not improved group. In SURMOUNT-4, 91.6 % of participants shifted at least one BMI category lower, with a mean BMI change from baseline of -10.47 kg/m^2^ in the improved group versus -5.20 kg/m^2^ in the not improved group (Table 1, Table 2). Furthermore, 45.3 % of participants treated with tirzepatide in SURMOUNT-1 and 64.2 % of participants treated with tirzepatide in SURMOUNT-4 improved by two or more BMI categories by the end of the treatment period, as compared to baseline.

**Table 2:** Shifts in BMI category from baseline to Week 72 (SURMOUNT-1) and baseline^a^ to Week 88 (SURMOUNT-4) in participants treated with tirzepatide.

Data were analyzed using the full analysis set from the mITT population, excluding data after discontinuation of the study drug. BMI shifts to a “decreased BMI category” or an “increased BMI category” are indicated in green and red, respectively.

^a^Baseline in SURMOUNT-4 is the lead-in baseline at Week 0.

^b^Data from the 10 mg and 15 mg tirzepatide dose groups were combined.

BMI = body mass index; mITT = modified intent-to-treat; MTD = maximum tolerated dose; N = number of participants in the given population

In contrast, a small percentage of participants treated with tirzepatide remained in the same BMI category (18.0 % in SURMOUNT-1 and 8.1 % in SURMOUNT-4), and an even smaller percentage shifted to a higher BMI category with tirzepatide treatment (0.2 % in SURMOUNT-1 and 0.3 % in SURMOUNT-4) (Table 2).

The proportions of participants in each BMI category at baseline and at the end of the treatment period are shown in Fig. 2. Notably, tirzepatide treatment was associated with achieving a BMI no longer in the obesity range (<30 kg/m^2^) in the majority of participants (57.2 % in SURMOUNT-1 and 69.3 % in SURMOUNT-4) (Fig. 2). Additionally, nearly one in four participants in SURMOUNT-1 (23.4 %) and one in three in SURMOUNT-4 (31.9 %) reached a normal BMI (<25 kg/m^2^) with tirzepatide treatment (Fig. 2).

In both studies, only sex (lower chances of improvement for males relative to females) and baseline BMI (lower chances of improvement with higher BMI) were significantly associated with BMI shifts following multivariate analysis (SURMOUNT-1: sex, p<0.001; baseline BMI, p<0.001; SURMOUNT-4: sex, p=0.006; baseline BMI, p<0.001).

### 3.3 Changes in cardiometabolic risk factors

Fig. 3 shows that in the SURMOUNT-1 trial, for all the cardiometabolic parameters shown, participants who shifted to a lower BMI category (defined as improved BMI category) also had a significant improvement from baseline in cardiometabolic risk factors. Participants from the SURMOUNT-4 trial in the improved BMI category demonstrated greater improvement in cardiometabolic risk factors, including waist circumference, fasting serum glucose, fasting insulin, triglycerides, high-density lipoprotein cholesterol (HDL-C), and very-low-density lipoprotein cholesterol, compared to those with no shift or those who shifted to a higher BMI category (not improved BMI category) (Fig. 4). In both SURMOUNT-1 and SURMOUNT-4, very-low-density lipoprotein cholesterol and triglycerides decreased over 31 % in the improved BMI category, and fasting insulin levels decreased over 53 % in both trials (Fig. 3, Fig. 4).

## 4 Discussion

In these post hoc analyses of the SURMOUNT-1 and SURMOUNT-4 trials, shifts from baseline in BMI category following tirzepatide treatment and their associations with changes in cardiometabolic risk factors in adults with overweight or obesity were noted. To our knowledge, this is one of the first studies to expand beyond traditional metrics such as weight loss in kilograms or percent and examine BMI category shifts and related changes in cardiometabolic risk factors occurring with obesity management medications in adults living with obesity and overweight.

Obesity is a known risk factor for cardiovascular disease, and weight reduction is associated with improvements in established causal cardiovascular disease risk factors, such as systolic blood pressure and blood lipids, and influences hemodynamic changes, low-grade inflammation, and prothrombotic changes, pathways relevant to multiple vascular outcomes [[12], [13], [14]]. In both SURMOUNT-1 and SURMOUNT-4, the majority of participants treated with tirzepatide (81.8 % and 91.6 %, respectively) shifted at least one BMI category lower (Table 2). Furthermore, a substantial proportion of participants treated with tirzepatide (45.3 % in SURMOUNT-1 and 64.2 % in SURMOUNT-4) improved by two or more BMI categories by the end of the treatment period (Table 2). These results are clinically relevant, as BMI reductions sufficient to shift participants into a lower, improved BMI category generally require substantial weight reduction.

Tirzepatide treatment was associated with reaching a BMI category no longer in the obesity range (<30 kg/m^2^) in the majority of participants (57.2 % in SURMOUNT-1 and 69.3 % in SURMOUNT-4) (Fig. 2). Further, nearly one in four participants in SURMOUNT-1 (23.4 %) and one in three participants in SURMOUNT-4 (31.9 %) reached a normal BMI (<25 kg/m^2^) with tirzepatide treatment (Fig. 2). Achieving a normal BMI has been shown to resonate with people living with obesity and overweight as a desired clinical goal; however, few studies have explored this outcome. Thus, future studies are warranted to better understand the durability of remaining in the normal BMI category and its related clinical benefit [15,16]. Conversely, our analyses also highlight a subgroup of participants who, despite 72–88 weeks of tirzepatide treatment, remained living with severe obesity, defined by the class III BMI category (>40 kg/m^2^) of which there were 4.8 % (N=332) in SURMOUNT-4 and 9.7 % (N=1884) in SURMOUNT-1 (Table 2). Those who did not shift, even if they lost weight, were at baseline generally of higher BMI and more likely to be male than those who did shift (Table 1). As one in four US Americans are projected to have severe (class III) obesity by 2030 [17], these findings underscore the need for continued—and potentially intensified—obesity management strategies to help this high-risk group achieve clinically meaningful outcomes.

Overall, we found that participants treated with tirzepatide in SURMOUNT-1 and SURMOUNT-4 who shifted to a lower BMI category generally experienced favorable changes in cardiometabolic measures (Fig. 3, Fig. 4). It is hypothesized that these changes could translate to meaningful reductions in overall cardiometabolic risk.

Our work extends the results of previous research in tirzepatide-treated participants with T2D, which showed that shifting to an improved BMI category was generally associated with numerical improvements in selected cardiometabolic parameters [18]. In adults with obesity, several studies have shown that obesity treatment reduces cardiovascular disease burden. For example, the SELECT trial (which enrolled overweight or obese patients with cardiovascular disease but without diabetes) demonstrated substantial weight reduction with high-dose semaglutide and a reduction in major cardiovascular events; however, the magnitude of weight loss did not directly correlate with semaglutide’s effects on major atherosclerotic cardiovascular event reduction [19]. In the recent SUMMIT trial, tirzepatide treatment was associated with a lower risk of a composite of cardiovascular death or worsening heart failure compared to placebo [5]. Whether the substantial weight reductions observed with tirzepatide treatment can impact morbidity and mortality in adults with obesity who are at risk of cardiovascular disease is currently being investigated in participants with obesity in the SURMOUNT-MMO trial (NCT05556512).

In the present analyses, tirzepatide treatment was associated with substantial reductions in blood pressure among individuals with overweight or obesity (Fig. 3, Fig. 4) [8,11,20]. A post hoc analysis of SURMOUNT-1 by Krumholz et al. [21] showed that blood pressure declined rapidly over the first 24 weeks of tirzepatide treatment and stabilized by Week 72, with significant reductions in both diastolic and systolic values. Overall, non-HDL-C appears to be a more accurate indicator of cardiovascular death risk than low-density lipoprotein cholesterol. Non-HDL-C captures more atherogenic lipid particles, as it contains very-low-density lipoprotein cholesterol or remnants which are more harmful than low-density lipoprotein cholesterol particles [22,23]. In a large cohort at low 10-year risk of atherosclerotic cardiovascular disease from the Cooper Center Longitudinal Study that was followed for over two decades, non-HDL-C ≥160 mg/dL was significantly associated with cardiovascular disease and coronary heart disease mortality [24]. Furthermore, compared to non-HDL-C levels below 130 mg/dL, all categories of non-HDL-C levels 130 mg/dL and above were associated with higher cardiovascular disease death rates [24]. The observed improvements in blood pressure regulation and lipid profile in our analyses offer additional beneficial effects of tirzepatide, alongside its observed effects on body weight reduction.

There are several important strengths and limitations of these analyses that should be noted. Participants in both SURMOUNT-1 and SURMOUNT-4 achieved significant and substantial weight loss, providing sufficient numbers to examine shifts in individual BMI categories as well as overall comparisons between improved and not improved categories. The difference in trial duration (72 weeks for SURMOUNT-1 and 88 weeks for SURMOUNT-4) also allowed for examination of how additional treatment might influence BMI category shifts and corresponding changes in cardiometabolic risk factors. However, the use of BMI categories and the identified shifts between them has limitations. Each category spans a five-point BMI range, meaning that participants near the upper end of a given category may experience considerable weight loss without moving into a lower category. In such cases, a lack of BMI shift does not imply absence of treatment effect—substantial improvements in cardiometabolic parameters may still be observed. This distinction is evident when examining the changes from baseline in these parameters within the improved and not improved groups compared to the static endpoint results (Fig. 3, Fig. 4). Additionally, while our analyses included participants with BMIs up to 45 kg/m^2^, they did not capture tirzepatide-treated individuals from the very high BMI group (≥60 kg/m^2^). This patient population has been reported to be rapidly growing and warrants further investigation for treatment [25]. Finally, the follow-up periods for these trials were 72 and 88 weeks, limiting inference for long-term cardiovascular outcomes. Longer-term studies could provide additional insights into the potential impact of BMI changes related to tirzepatide on long-term cardiovascular health.

## 5 Conclusion

In these post hoc analyses of SURMOUNT-1 and SURMOUNT-4, the majority of participants treated with tirzepatide shifted to lower improved BMI categories in both trials. This shift was accompanied by significant improvements in cardiometabolic risk factors, including waist circumference, HbA1c, blood pressure, and lipid profile. These findings suggest that tirzepatide treatment may contribute to improved future cardiovascular outcomes. Further studies are needed to better understand the clinical significance of long-term treatment with obesity management medications; relevant outcomes trials are currently underway.

## Author agreement

All authors have seen and approved the final version of the manuscript. The authors confirm that the manuscript represents their original work, has not been published previously, and is not under consideration for publication elsewhere.

## Glossary of field specific terms

Efficacy analysis set; Data obtained during treatment period from the modified intent-to-treat population, excluding data after stopping study drug.

MTD = maximum tolerated dose; The highest dose of a drug or treatment that does not cause unacceptable side effects. mITT = modified intent-to-treat; All randomly assigned participants who are exposed to at least one dose of study drug.

## CRediT authorship contribution statement

**Naveed Sattar:** Writing – review & editing, Methodology. **Clare J. Lee:** Writing – review & editing. **Reshmi Srinath:** Writing – review & editing. **Philip R. Schauer:** Writing – review & editing. **Hui Wang:** Writing – review & editing, Methodology, Formal analysis. **Beverly Falcon:** Writing – review & editing. **Xuanyao He:** Writing – review & editing. **Adam Stefanski:** Writing – review & editing, Investigation. **Arian Plat:** Writing – review & editing, Writing – original draft, Formal analysis, Methodology.

## Declaration of competing interest

The authors declare the following financial interests/personal relationships which may be considered as potential competing interests:

Naveed Sattar reports a relationship with AstraZeneca that includes: consulting or advisory. Naveed Sattar reports a relationship with Boehringer Ingelheim that includes: consulting or advisory and funding grants. Naveed Sattar reports a relationship with Eli Lilly and Company that includes: consulting or advisory. Naveed Sattar reports a relationship with Hanmi Pharmaceuticals that includes: consulting or advisory. Naveed Sattar reports a relationship with Novo Nordisk that includes: consulting or advisory. Naveed Sattar reports a relationship with Novartis that includes: consulting or advisory. Naveed Sattar reports a relationship with Sanofi that includes: consulting or advisory. Naveed Sattar reports a relationship with Pfizer that includes: consulting or advisory. Naveed Sattar reports a relationship with Amgen that includes: consulting or advisory. Phillip R Schauer reports a relationship with GI Dynamics that includes: board membership. Phillip R Schauer reports a relationship with Heron that includes: board membership. Phillip R Schauer reports a relationship with Eli Lilly and Company that includes: board membership. Phillip R Schauer reports a relationship with Regeneron that includes: board membership. Philip R Schauer reports a relationship with Ethicon that includes: consulting or advisory and funding grants. Philip R Schauer reports a relationship with Medtronic that includes: consulting or advisory and funding grants. Phillip R Schauer reports a relationship with Novo Nordisk that includes: consulting or advisory. Phillip R Schauer reports a relationship with National Institutes of Health that includes: funding grants. Phillip R Schauer reports a relationship with SEHQC LLC that includes: equity or stocks. Phillip R. Schauer reports a relationship with Medifilx that includes: equity or stocks. Phillip R Schauer reports a relationship with Metabolic Health International LTD that includes: equity or stocks. Reshmi Srinath reports a relationship with Dexcom that includes: funding grants. Reshmi Srinath reports a relationship with Novo Nordisk that includes: consulting or advisory and funding grants. Reshmi Srinath reports a relationship with Pfizer that includes: funding grants. Reshmi Srinath reports a relationship with Kintai that includes: consulting or advisory and funding grants. Reshmi Srinath reports a relationship with Ionis that includes: consulting or advisory and funding grants. Reshmi Srinath reports a relationship with Astra Zeneca that includes: funding grants. Reshmi Srinath reports a relationship with Sanofi that includes: consulting or advisory. Reshmi Srinath reports a relationship with Schocia that includes: consulting or advisory. Reshmi Srinath reports a relationship with ReDesign Health that includes: equity or stocks. Reshmi Smith reports a relationship with Zafgen that includes: equity or stocks and funding grants. Clare J Lee reports a relationship with Eli Lilly and Company that includes: employment and equity or stocks. Beverly Falcon reports a relationship with Eli Lilly and Company that includes: employment and equity or stocks. Xuanyao He reports a relationship with Eli Lilly and Company that includes: employment and equity or stocks. Adam Stefnaski reports a relationship with Eli Lilly and Company that includes: employment and equity or stocks. Arian Plat reports a relationship with Eli Lilly and Company that includes: employment and equity or stocks. Hui Wang reports a relationship with Eli Lilly and Company that includes: employment. If there are other authors, they declare that they have no known competing financial interests or personal relationships that could have appeared to influence the work reported in this paper.
